# Inference of haplotypic phase and missing genotypes in polyploid organisms and variable copy number genomic regions

**DOI:** 10.1186/1471-2105-9-513

**Published:** 2008-12-01

**Authors:** Shu-Yi Su, Jonathan White, David J Balding, Lachlan JM Coin

**Affiliations:** 1 Department of Epidemiology and Public Health, Imperial College, London, W2 1PG, UK; 2 National Institute of Agricultural Botany, Huntingdon Road, Cambridge, CB3 OLE, UK

## Abstract

**Background:**

The power of haplotype-based methods for association studies, identification of regions under selection, and ancestral inference, is well-established for diploid organisms. For polyploids, however, the difficulty of determining phase has limited such approaches. Polyploidy is common in plants and is also observed in animals. Partial polyploidy is sometimes observed in humans (e.g. trisomy 21; Down's syndrome), and it arises more frequently in some human tissues. Local changes in ploidy, known as copy number variations (CNV), arise throughout the genome. Here we present a method, implemented in the software polyHap, for the inference of haplotype phase and missing observations from polyploid genotypes. PolyHap allows each individual to have a different ploidy, but ploidy cannot vary over the genomic region analysed. It employs a hidden Markov model (HMM) and a sampling algorithm to infer haplotypes jointly in multiple individuals and to obtain a measure of uncertainty in its inferences.

**Results:**

In the simulation study, we combine real haplotype data to create artificial diploid, triploid, and tetraploid genotypes, and use these to demonstrate that polyHap performs well, in terms of both switch error rate in recovering phase and imputation error rate for missing genotypes. To our knowledge, there is no comparable software for phasing a large, densely genotyped region of chromosome from triploids and tetraploids, while for diploids we found polyHap to be more accurate than fastPhase. We also compare the results of polyHap to SATlotyper on an experimentally haplotyped tetraploid dataset of 12 SNPs, and show that polyHap is more accurate.

**Conclusion:**

With the availability of large SNP data in polyploids and CNV regions, we believe that polyHap, our proposed method for inferring haplotypic phase from genotype data, will be useful in enabling researchers analysing such data to exploit the power of haplotype-based analyses.

## Background

Haplotype analysis plays an important role in the mapping of disease genes where it has been shown to be more powerful than single-marker analysis [1-3] and provides finer localisation of causative mutations [4]. Haplotype-based methods may also be used to infer aspects of population history, such as the effects of positive selection [5] and recombination events [6].

Experimental methods for determining haplotypic phase are available, but are prohibitively expensive for large-scale studies [7]. Consequently statistical inference of haplotypic phase has become a popular alternative. A variety of maximum-likelihood [8-10] and Bayesian [11] methods has been developed for diploid genotypes. Currently, two popular methods are PHASE [12], based on the coalescent model, and fastPhase [13], based on an ancestral haplotype-clustering model. While preparing this article, we became aware that a software, SATlotyper [14], was developed for inferring haplotypic phase in polyploids using a parsimony based approach, but we found it to be incapable of analysing the datasets in our simulation study, which consist of hundreds of markers and hundreds of individuals.

Polyploidy is common in plant species, for example potato (*Solanum tuberosum*, 4n) and sugar beet (*Beta vulgaris*, 2n, 3n, 4n), and also occurs in animals such as goldfish (*Carassius auratus*, 4n). As these examples illustrate, tetraploidy (4n) is most common [15] but other ploidy levels occur. Partial polyploidy arises in humans, occasionally at the level of entire chromosomes (e.g. trisomy 21; Down's syndrome). Within-chromosome changes in ploidy, known as copy number variations (CNV), occur frequently throughout the genome and span intervals up to many megabases. Some human tissues (e.g. heart and liver) are known to frequently show variable ploidy, as do cancer cells. Our focus here is on autopolyploid populations such as tetraploid potato. In contrast, for allopolyploids such as wheat, pairs of chromosomes with distinct origins are analyzed as diploid data using markers that are specific to each pair.

There is currently much interest in kinship as a confounding factor in association mapping [16,17] and kinship can be estimated more accurately from phased than from unphased genotypes [18]. The study of linkage disequilibrium (LD) provides a basis both for mapping disease genes and for the investigation of evolutionary history. Some LD-based studies have been conducted in polyploid species [19,20]. To overcome the problems of inferring phase, most of this work was conducted on homozygous individuals from inbred populations. However, since the effects of inbreeding depression often prevent the achievement of widespread homozygosity, this approach is often limited to small regions of chromosomes. It may also be inappropriate to make generalisations about the evolution of an out-breeding species using data from artificially created inbreds [21]. Another way to conduct LD association analyses in polyploids is to treat the polyploid genotypes as diploids, which can generate biased estimates. It is clear, therefore, that there is much information in polyploid genomes that is currently hidden from us due to the lack of phased genotype data.

Here we propose a method, implemented in the software polyHap, for the inference of haplotypic phase and missing observations from polyploid genotype data. Our method is a generalisation of the approach developed in [3] and employs a hidden Markov model (HMM) to infer an ancestral cluster for each haplotype at each marker, reflecting the idea that similar haplotypes are likely to have descended from the same ancestral haplotype. The ancestral cluster allocations of an individual are highly correlated across tightly-linked loci, reflecting limited recombination since the common ancestors. PolyHap first learns the ancestral clusters from genotype data jointly for all individuals, then infers phased sets of ancestral clusters for each individual, and hence the underlying haplotypes.

PolyHap is designed for use with SNP genotype data, where the marker order is known. With genetic or physical maps of plant genomes becoming increasingly available (including that of potato [20,22]) and with increasing numbers of CNV regions identified and improving technology for genotyping copy number polymorphisms (CNP), we believe that polyHap provides a timely addition to the geneticist's toolkit.

## Results and discussion

### Results

Due to the limited availability of phased SNP data from polyploid species, we evaluated the performance of polyHap by randomly combining human male X-chromosome haplotypes from the WTCCC to create datasets of artificial diploid, triploid and tetraploid genotypes (see methods). For diploids we compare with fastPhase which is a well established method and of comparable computational speed to polyHap. For triploids and tetraploids, we attempted to compare with SATlotyper, but we found that the scale of our simulated dataset was not computationally feasible for SATlotyper. To evaluate the imputation error rate, 1% of all genotypes were set as missing, and we report in Table 1 the proportions of missing genotypes that were assigned incorrect genotypes for polyHap and (diploids only) fastPhase. With eight ancestral haplotype clusters in diploids, polyHap had an imputation error rate of 3.9% after one repetition of the EM algorithm (see methods), falling to 3.8% after ten repetitions, compared with 5.0% and 4.6% for fastPhase. With 20 clusters, the error rate of 4.4% (4.3%) for fastPhase with one (ten) repetitions remained higher than for polyHap with eight clusters. For triploid data, polyHap's error rate increased to 5.6% (4.7%), and for tetraploid data it was 7.3% for one repetition (due to computational demands, only one repetition was performed on the tetraploid dataset).

**Table 1:** Imputation Error Rates

	diploid		triploid		tetraploid
	
method	I = 1	I = 10	I = 1	I = 10	I = 1
polyHap:					
*z *= 8	0.039	0.038	0.056	0.047	0.073
*z *= 6	0.040	0.045	0.058	0.060	0.087
*z *= 4	0.049	0.044	0.077	0.065	0.106
*z *= 2	0.101	0.085	0.144	0.114	0.194
fastPhase:					
*z *= 20	0.044	0.043			
*z *= 10	0.046	0.045			
*z *= 8	0.050	0.046			

I is the number of repetitions of the EM algorithm.

**Figure 1:**
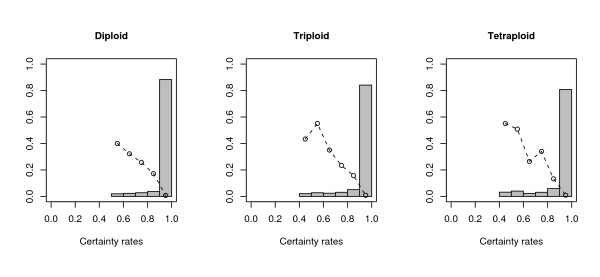
**Histogram of certainty scores and imputation error rate in each bin**. The histogram shows the distribution of certainty scores for imputations of missing genotypes, when polyHap was run with eight ancestral clusters and one repetition of the EM algorithm. The circles indicate average imputation error rates within each histogram bin.

PolyHap also provides a measure of certainty between zero and one for each imputed genotype. Figure 1 shows a histogram of the distribution of certainty scores, and the average imputation error rate in each histogram bin, after running polyHap with eight clusters and one repetition. For all ploidies, more than 80% of predicted genotypes have certainty > 0.9, and the imputation error rates are < 1% in this bin. More generally, the dashed curves in Figure 1 lie close to the line *y *= 1 - *x*, indicating that our certainty score is approximately the probability that the imputation is correct.

The switch error rate for an individual is defined as the minimum number of switches required to reconstruct its true haplotypes from the phased haplotypes provided by polyHap, divided by number of heterozygous markers minus one. Average switch error rates are reported in Table 2. With eight ancestral haplotype clusters, polyHap had an error rate of 7.8% (6.8%) for one (ten) repetitions respectively, compared with 9.3% (7.8%) for fastPhase. With 20 clusters, fastPhase had a switch error rate of 8.7% (7.1%), still greater than for polyHap with eight clusters. For triploid data, polyHap's error rate increased to 12.7% (11.4%) and for tetraploid data, it was 17.5% for one repetition.

**Table 2:** Switch Error Rates

	diploid		triploid		tetraploid
	
method	I = 1	I = 10	I = 1	I = 10	I = 1
polyHap:					
*z *= 8	0.078	0.068	0.127	0.114	0.175
*z *= 6	0.085	0.074	0.132	0.117	0.239
*z *= 4	0.098	0.082	0.147	0.127	0.263
*z *= 2	0.153	0.119	0.241	0.186	0.323
fastPhase:					
*z *= 20	0.087	0.071			
*z *= 10	0.098	0.076			
*z *= 8	0.093	0.078			

I is the number of repetitions of the EM algorithm.

**Figure 2:**
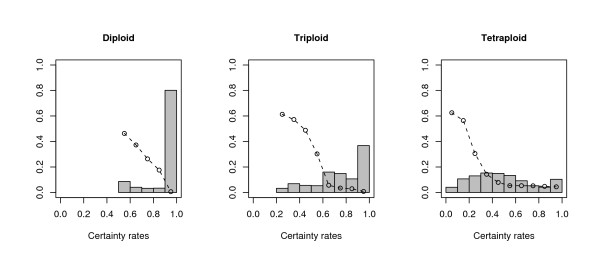
**Histogram of certainty scores and switch error rate in each bin**. The histogram shows the distribution of phasing certainty scores, averaged over individuals, for the same polyHap runs as in Figure 1. The circles indicate average switch error rates within each histogram bin.

PolyHap also provides a certainty measure for the phase assignment. Figure 2 shows the distribution of certainties over ten bins, as well as the average switch error rate in each bin for a run of polyHap with eight clusters and one repetition. For all ploidies, there is a decreasing relationship between certainty of phase assignment and switch error rate. For diploids, the certainties above 50% are well calibrated with the switch error rate. Moreover, 80% of phase estimates have certainty > 0.9, and the switch error rate is < 1% in this bin. For triploids, ~75% of phase estimates have certainty > 0.7, and average switch error rate is < 6% in each of these bins. For tetraploid data, ~50% of phase estimates have certainty > 0.5, and the average switch error rate is < 8% in each of these bins.

**Figure 3:**
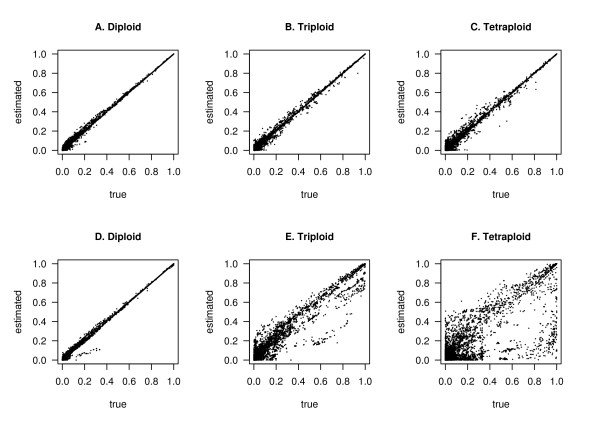
**Comparison between estimated and true*****r***^**2**^. Estimated versus true *r*^2 ^values for diploids, triploids, and tetraploids, respectively for all pairs of markers separated by < 500 Kb. The estimated *r*^2 ^in A-C is calculated from haplotypes inferred using the same polyHap runs (with eight clusters and 1 repetition) as in Figure 2, while in D-F is calculated from haplotypes inferred using fastPhase (with 20 clusters and 10 repetitions) by treating the polyploid genotypes as diploid. The Pearson correlation coefficients between estimated and true *r*^2 ^for A-F are 0.999, 0.997, 0.996, 0.998, 0.975 and 0.840 respectively.

To assess the effect of haplotype inferences on inferences about LD, we compared *r*^2 ^values calculated from inferred haplotypes (estimated *r*^2^) with those from the simulated haplotypes (true *r*^2^). Panels A-C of Figure 3 shows that *r*^2 ^estimated via polyHap is highly correlated with the true *r*^2^, even for tetraploids. Similarly in Panel D, *r*^2 ^estimated via fastPhase and the true *r*^2 ^are highly correlated. Panels E and F show that existing methods based on treating polyploid genotypes as diploid lead to imprecise estimates of *r*^2^.

We applied our method to a real dataset which consists of 12 SNPs from 19 tetraploid potatoes with known haplotypic phase obtained from the laboratory, and compared our results with those from SATlotyper. PolyHap had an average switch error rate of 6.6% with eight ancestral haplotypes for ten repetitions, while SATlotyper had error rate of 8.8%. Also, among 17 samples (excluding two samples having homozygous genotypes), 15 samples were phased correctly for the whole sequence by polyHap, compared with 13 samples by SATlotyper.

The computing time of polyHap with eight clusters and one repetition for a dataset of 501 markers and 500 (488, 300) individuals for diploid (triploid, tetraploid) was approximately 0.5 (4, 30) hours. The corresponding computing time for fastPhase with 20 clusters and one repetition (only for diploid) was approximately 7 minutes. Although fastPhase with one repetition was fast, the results were less precise than those from polyHap. Attempting to run SATlotyper on our dataset of 501 markers and 300 tetraploid individuals generated an error due to excess memory requirement on a 16 GB computer (the same as was used to run polyHap). The problem persisted when we reduced the number of markers to 50. Reducing it further to 25 markers (still with 300 tetraploid individuals) allowed the program to run but it failed to generate a result after 72 hours.

## Discussion

We present the polyHap software for inferring haplotypes from polyploid genotype data with some missing observations, using a HMM similar to that underlying fastPhase [13] and HINT [23] for diploid data. The optimal number of ancestral clusters is typically unknown, and we have considered 2,4,6 and 8 clusters, finding in general that more clusters provide better inferences but at increased computational cost. Our approach does not require pre-defined haplotype blocks nor a sliding window scheme to define haplotype boundaries. Although we focus on diallelic markers here, polyHap could be adapted to allow for multi-allelic markers.

The results from our evaluation of polyHap are encouraging: using eight ancestral clusters we obtained imputation error rates of 4.7% and 7.3%, and switch error rates of 11% and 18%, for triploids and tetraploids respectively. For diploid data, we obtained lower imputation and switch error rates for polyHap than for fastPhase. Phasing using polyHap generated a considerable improvement in the estimation of *r*^2 ^from polyploid data over estimates based on treating polyploids as diploids. The certainty values generated by polyHap are approximately well-calibrated as error probabilities for imputation of missing genotypes, and provide a useful guide to the quality of phase inferences.

We have shown that polyHap outperforms SATlotyper on a tetraploid dataset which was experimentally haplotyped. The switch error rates are 6.6% for polyHap compared to 8.8% for SATlotyper and more samples are phased correctly by polyHap (88%) than SATlotyper (76%). Although this potato dataset is small, our results strongly suggest that polyHap is at least as good as SATlotyper for phasing a short region. Moreover, polyHap is able to phase genotypes in a long region with many SNPs whereas SATlotyper is limited to analysing a relatively small number of SNPs.

PolyHap also supports datasets containing individuals of different ploidy, but not variable ploidy within an individual. However, in the latter case separate runs of polyHap can be applied over regions where within-individual ploidy is constant. In some situations, phased haplotypes are available for some individuals (e.g. from a study of inbreds or other laboratory process). PolyHap can exploit the extra information about haplotype structure from any available phased individuals; the resulting reduction in error rates will vary depending on sample size and other factors.

In addition to imputing completely missing genotypes, polyHap also supports a scenario in which the genotype is partially known, for example at dominant markers in which only the presence or absence of a particular allele is known. Our model, incorporating this partial information from available alleles, is expected to give better inference than the conventional approach which ignores the partially available alleles and considers partially missing data as completely missing.

## Conclusion

Knowledge of phase allows powerful haplotype-based methods to be exploited in genetic analyses. The inference of phase in the diploid case is well established and the advantages of inferred haplotypes are now widely appreciated. Polyploid organisms present greater challenges and, until now, phase has been difficult to determine routinely unless detailed pedigree information is available.

Using our polyHap software, the phased genotypes of triploid and tetraploid organisms can now be inferred from samples of unrelated individuals. Importantly, the quality of the inferences can be measured and allowed for in subsequent analyses. Thus the quality of subsequent inferences can be assessed, reducing the risk of overconfident extrapolations from imperfect data.

In terms of both switch and imputation error rates in the diploid case, polyHap was superior in our simulation study to a well-established software, and offers good accuracy at higher ploidy. As a demonstration of the utility of the approach, we show good agreement in terms of LD (measured as *r*^2^) between inferred and true data.

With increasing marker densities and improving genetic and physical maps in many polyploid species, together with increasing information about, and recognition of the potential importance of CNV regions in humans and other species, we believe that polyHap will prove to be a timely addition to the geneticists toolkit.

## Methods

We will first briefly describe the construction of the polyploid HMM, which is a development of our previous work; see [3] for further details. We then describe the sampling algorithm for inferring missing genotypes and haplotypic phase as well as for calculating a certainty for each estimate.

Our notation is similar to that of [3], where *g *= (*g*_1_, *g*_2_....,*g*_*M*_) are the genotypes of *M *SNPs for an individual; *N *is the ploidy and *g*_*m *_= {*g*_*m*1_,...*g*_*mN*_} is an unordered list of the individual's alleles at marker *m*. Each allele is assumed to have been drawn from one of *z *ancestral clusters. Also, *s*_*m *_= {*s*_*m*1_,...,*s*_*mN*_} and ${s^{\prime }}_{m}$ = [*s*_*m*1_,...,*s*_*mN*_] are the unordered and ordered lists of clusters at marker *m*, respectively. We write *π*(${s^{\prime }}_{m}$) = [*s*_*mπ*(1)_,...,*s*_*mπ*(*N*)_] for a permutation of ${s^{\prime }}_{m}$, and ∏(*s*_*m*_) for the set of all such permutations.

### Transition probabilities for the polyploid HMM

Transitions in the HMM correspond to recombination events at which an individual's haplotype switches the cluster from which it is considered to have descended. First we define the transition probability in a haploid HMM from clusters *k*_*n *_to *l*_*n *_between markers *m*-1 and *m *by:

(1) $$p(s_{mn}=l_{n}|s_{(m-1)n}=k_{n})=\{\begin{array}{ll} (1-J_{m})+J_{m}\alpha_{ml_{n}} & l_{n}=k_{n}\\ J_{m}\alpha_{ml_{n}} & l_{n}\neq k_{n,} \end{array}$$

where *J*_*m *_is the probability of a jump occurring at marker *m*-1, and the probability that a jump results in cluster *l*_*n *_is $\alpha_{ml_{n}}$, irrespective of the current cluster. For tightly-linked markers, *J*_*m *_is small so that cluster changes occur infrequently, but are allowed between any pair of markers. Gene conversion events are not modelled explicitly but can be accommodated in our model by two proximal recombination events. Based on this haploid model, the transition probability between ordered lists of clusters [*k*_1_,...,*k*_*N*_] and [*l*_1_,...,*l*_*N *_] is given by

(2) $$p({s^{\prime }}_{m}=[l_{1},...,l_{N}]|{s^{\prime }}_{\left(m-1\right)}=[k_{1},...,k_{N}])=\prod_{n=1,...,N}p(s_{mn}=l_{n}|s_{(m-1)n}=k_{n}),$$

and between unordered lists of clusters it is

(3) $$p(s_{m}=\{l_{1},...,l_{N}\}|s_{\left(m-1\right)}=\{k_{1},...,k_{N}\})=\sum_{\pi \in \Pi (s_{m})}\prod_{n=1,...,N}p(s_{mn}=l_{\pi (n)}|s_{(m-1)n}=k_{n}).$$

### Emission probabilities for the polyploid HMM

The relation between hidden cluster and observed genotype data is modelled by emission probabilities. As for the transition probability, the emission probability of the polyploid HMM can be derived from a haploid model. For generality, we assume multiallelic markers with alleles *h *∈ {0,...,*H*}. Denote *θ*_*ml*_(*h*) the emission probability of allele *h *in a haploid model at marker *m *from cluster *l*. We then obtain the emission probability of a genotype given an unordered list of ancestral haplotypes *s*_*m *_by

(4) $$p(g_{m}=\{h_{1,}...,h_{N}\}|s_{m}=\{l_{1},...,l_{N}\}))=\sum_{\pi \in \Pi (g_{m})}\prod_{n=1,...,N}p(g_{mn}=h_{\pi (n)}|s_{mn}=l_{n})=\sum_{\pi \in \Pi (g_{m})}\prod_{n=1,...,N}\theta_{ml_{n}}(h_{\pi (n)}),$$

If *g*_*m *_is completely missing for an individual at marker *m*, we set *θ*_*ml*_(*h*) to be uniform over all alleles. If *g*_*m *_is partially missing, we set *θ*_*ml*_(*h*) to be uniform over alleles that are consistent with the observed genotype at marker *m*.

We use Dirichlet priors on all of our parameters. We let *θ*_*ml *_~ Dirichlet(*u*_*θ*_**m**_*θ*_), where **m**_*θ *_is the uniform vector with each element equal to 1/*H*, and *α*_*m*_. ~ Dirichlet(*u*_*α*_**m**_*α*_) where **m**_*α *_is the uniform vector with each element equal to 1/*z*. We also let $J_{m}\sim \text{Beta}(u_{J}(1-e^{-d_{m}r}),u_{J}e^{-d_{m}r})$ where *d*_*m *_is the physical distance between consecutive markers and *r *= 10^-8 ^per base pair, reflecting the background recombination rate. We use *u*_*θ *_= *u*_*α *_= 1 and *u*_*J *_= 10^5 ^for initialisation of the EM algorithm and *u*_*θ *_= *u*_*α *_= *u*_*J *_= 0.1 for the maximisation step.

Although our HMM has many parameters, *J*_*m *_at each marker and *α*_*ml *_and *θ*_*hml *_at each hidden cluster and each marker, approximate posterior mode estimates are readily obtained using an expectation maximisation (EM) algorithm, which in this setting is usually referred to as the Baum-Welch algorithm.

### Estimating genotypes and haplotypes from the HMM

After each repetition of the Baum-Welch EM algorithm which consists of a default 25 iterations, we obtain estimates of the parameters of the HMM which approximate a local mode of their joint posterior distribution. For each individual, we then run the forward-backward algorithm using these parameter values to obtain a 'trace' matrix of the probability, conditional on the model being in state *l *at marker *m*, of having arrived from state *k *at marker *m*-1. We can then sample from the posterior distribution over state-paths conditional on the non-missing genotype data of a given individual, by starting at the 'end' state, position *M*+1, and moving back towards marker 1, sampling the state at the previous marker using the elements of this trace matrix. Each state in this state-path consists of an unordered list of ancestral haplotype clusters. At each marker we use (2) to sample a state of ordered haplotype clusters from these unordered haplotype clusters. We then sample from the probability distribution of ordered lists of alleles which are consistent with the genotype data at this marker, given the ordered list of haplotype clusters. In the case of missing genotype data, all possible ordered lists of alleles are considered. As we are reconstructing ordered lists of alleles, we obtain the *N *haplotypes (recall that the ploidy is *N*).

A pre-specified number (e.g. 100) of haplotype assignments is calculated, as described above, for each of a number (e.g. 10) of repetitions of the EM algorithm. From this we obtain a number of haplotype assignments (e.g 1000 = 10 × 100) for each individual. At markers with missing genotype data, the genotype that is sampled most often is reported as the imputed genotype, and the fraction of times it is sampled is reported as the 'certainty' of this estimate. Because we consider only a small number (e.g. 10) of local modes of the posterior distribution for the HMM parameters, our certainty value is not the probability of the imputed genotype under our model, which would require integration over the posterior distribution, but it may serve as a reasonable approximation to this probability.

The algorithm to calculate the most likely phase assignment for an individual on the basis of the set of sampled haplotypes proceeds from left to right. Consider a heterozygous marker, say *m*. If there is no missing data, all samples from the HMM have the same genotype at *m*; otherwise, we only consider samples which have the most common genotype. We assume that the haplotype configuration for all of the samples from the HMM is the same up to and including marker *m*-1, which is trivially true of the first heterozygous marker. We count the number of times each haplotype configuration (up to and including marker *m*) is observed, and return the configuration that is observed the most often as the phase assignment up to and including marker *m*; and the fraction of times this is observed as the 'certainty' of this configuration. We then go through each of the samples and as necessary introduce a switch between markers *m*-1 and *m *so that each of the samples now has the same haplotype configuration up to and including marker *m *as the reported configuration. At the stage of counting haplotype configurations, we note that it is only necessary to count as far back as to identify *N *different haplotypes for each sample.

## Material and simulation

### Simulated dataset

Haplotype data were obtained from a 6.4 Mb non pseudo-autosomal region of the X-chromosome (34,135,863 to 40,527,829 bp) genotyped by the WTCCC [24]. We included 1464 males from the 1958 British Birth Cohort and the UK Blood Service Control Group, after removing eight males showing high levels of heterozygosity. We then removed 243 SNPs on the whole X-chromosome which showed any heterozygosity. We analysed all 501 sites reported by the WTCCC, of which only 468 were polymorphic in our sample and 426 had minor allele frequency > 1%. We randomly combined the the X-chromosomes, first into pairs to create 500 diploid genotypes, then into triples and then quadruples, to create 488 triploid and 300 tetraploid genotypes.

### Experimentally haplotyped tetraploid dataset

The dataset, described in [14], consists of experimentally determined haplotypes at the *BA213c14t7 *locus with 12 SNP sites from 19 tetraploid individuals (potato). In [14], 590 amplicon derived clones were sequenced over a 500 bp region, and 10 distinct haplotypes were identified among those 19 samples. The most probable genotype for each sample was then determined based on the observed haplotypes and the frequency of each haplotype sequence (see detail in [14]). We analysed these genotypes using polyHap and SATlotyper, and assessed the performance of these two softwares by comparing the computed haplotypes with experimentally determined haplotypes.

### Switch error rate

**Figure 4:**
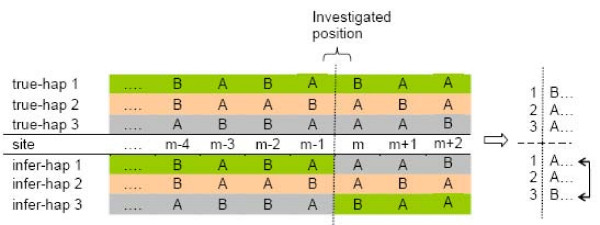
**Identifying a switch error in the triploid case**. In general we require at least *N *= 3 polymorphic sites to determine a phasing error, but because we can assume that the ordered haplotypes are correct up to and including marker *m–*1, we can see immediately that there is a switch at position *m *between haplotypes 1 and 3.

The switch error rate for each individual is defined as *ψ*/(*n*-1), where *n *denotes the number of heterozygous sites for that individual and *ψ *is the minimal number of switches needed to recover the true haplotypes. We assumed that at most one switch could occur between consecutive heterozygous sites. For each individual, we determined if there was a switch by comparing the inferred haplotypes to the true haplotypes. If a discrepancy is identified at a heterozygous marker *m*, a switch error is counted (e.g. Figure 4) and a switch is introduced in the inferred haplotypes to ensure that it matches the true haplotypes up to marker *m*. To identify a discrepancy, it is only necessary to compare haplotype sets as far back as to distinguish *N *distinct preceding haplotypes (N is the ploidy), which in diploids requires looking back to the previous heterozygous marker only.

### Assessment of r^2^

A commonly used statistic for measuring LD between two diallelic markers is the squared correlation coefficient, *r*^2^. To assess the effect of phasing errors on the resulting estimates, we compared *r*^2 ^values calculated from inferred haplotypes (estimated *r*^2^) with those from the simulated haplotypes (true *r*^2^). In some polyploid studies [20], multiple sets of chromosomes have been considered as diploid to measure LD, due to the limitations imposed by the markers used and the lack of phase information. To assess the effect of this approximation, we calculated *r*^2 ^via fastPhase by analysing polyploid genotypes as if they were diploid. For example, the tetraploid genotypes AAAB, AABB, and ABBB are all regarded as AB, while AAAA and BBBB are represented as AA and BB respectively.

## Availability and requirements

PolyHap is written in Java language and distributed as a Java jar file. It requires Java Runtime Environment with version 1.6.0_01 or later. The software can be executed from the command line or the supplied batch file under Unix like operating system. PolyHap is distributed under the GNU GENERAL PUBLIC license for non-commercial use only and can be downloaded from: .

## Authors' contributions

SS carried out the analysis, wrote the initial draft of the paper and jointly developed the polyHap software with LJMC. JW provided advice, and corrected sections of the paper. DJB oversaw the project with LJMC and edited the manuscript. LJMC instigated the project; jointly oversaw the project with DJB; jointly developed the software with SS, and edited the paper.
